# Assessment of the prevalence and risk factors of ophthalmoplegia among diabetic patients in a large national diabetes registry cohort

**DOI:** 10.1186/s12886-016-0272-7

**Published:** 2016-07-22

**Authors:** Eman S. Al Kahtani, Rajiv Khandekar, Khalid Al-Rubeaan, Amira M. Youssef, Heba M. Ibrahim, Ahmed H. Al-Sharqawi

**Affiliations:** King Khalid Eye Specialist Hospital, King Saud University, Riyadh, Saudi Arabia; Strategic Centre for Diabetes Research, King Saud University, Riyadh, Saudi Arabia; University Diabetes Centre, King Saud University, P.O. Box 18397, Riyadh, 11415 Saudi Arabia

**Keywords:** Ophthalmoplegia, III cranial nerve palsy, IV cranial nerve palsy, VI cranial nerve palsy, Diabetes mellitus

## Abstract

**Background:**

There are limited data on the epidemiology and risk factors of ophthalmoplegia among diabetic patients. This study aims to determine the prevalence and important risk factors related to ophthalmoplegia among diabetic patients.

**Methods:**

This is an observational registry-based study using the Saudi National Diabetes Registry (SNDR) database to select diabetic patients regardless of their diabetes type. A total of 64,351 Saudi diabetic patients aged more than 18 years and registered in SNDR between January 2000 and December 2010 were analyzed to identify ophthalmoplegic cases. Demographic, clinical, and biochemical parameters were studied and STROBE guidelines were used to design and report the results of this study.

**Results:**

The overall prevalence of ophthalmoplegia cases was 0.32 %, further distributed into: 53.11 %, 36.36 %, and 2.8 % for cranial nerves VI, III, IV palsies respectively. Ophthalmoplegic cases were predominantly type 2 diabetic males with older age and longer diabetes duration. The most important and significant risk factors were age ≥ 45 years, diabetes duration ≥ 10 years, male gender and presence of retinopathy and nephropathy.

**Conclusions:**

Ophthalmoplegia is a rare entity associated mainly with type 2 diabetes. Clinicians have to consider its risk factors when screening or planning for prevention of this condition.

## Background

Ophthalmoplegia is a rare entity seen in patients with diabetes mellitus and is associated with great patients’ anxiety and may appear as a serious diagnostic and therapeutic medical challenge. Ophthalmoplegia secondary to cranial nerve III, IV, and VI palsies in diabetic patients is considered to be a form of microvascular cranial nerve palsy involving small vessels atherosclerotic changes, however, full recovery within 12 weeks after the onset of symptoms is the usual outcome [1–3].

Despite extensive studies on the epidemiology of diabetic neuropathy in general, there is a relative paucity of knowledge regarding factors associated with ophthalmoplegia in patients with diabetes. Most of the published studies available in the literature have been generated from tertiary referral centres rather than general diabetic population [4, 5].

In a retrospective large Caucasian diabetic population follow up, mainly type 2 diabetes, 0.4 % were hospitalized with ophthalmoplegia [2], while a Japanese study reported the prevalence of ophthalmoplegia among diabetic patients to be 0.97 % which is ten times more than non-diabetic patients at 0.13 % [6]. Among Saudi diabetic patients, VI and III cranial nerves palsies were found to be the most frequently affected cranial nerves [7]. Ophthalmoplegia in diabetic patients are more frequently seen in older individuals with a long duration of diabetes [6]. Other risk factors are chronic diabetes micro and macroangiopathies namely: retinopathy, nephropathy, neuropathy and major vessel diseases [8].

Diabetes registry is a valuable source of clinical data that can be used to study rare conditions associated with diabetes. The Saudi National Diabetes Registry (SNDR) hosts large number of diabetic patients with different types of diabetes and a wide age spectrum. Over the past 14 years, this registry has collected information from a wide range of diabetic patients suffering from different cranial nerve palsies. Utilizing this registry, we performed a cross sectional prevalence study for ophthalmoplegia secondary to cranial nerves III, IV, and VI palsies and its related risk factors for patients who are older than 18 years of age regardless of their diabetes type.

## Methods

### Study population

This is an observational registry-based study using the data collected from patients’ hospital files for a specially designed electronic web-based data system that includes demographic data and diabetes related clinical and biochemical parameters in the Kingdom of Saudi Arabia. The design and development of the web-based SNDR has been explained in a previously published paper [9]. SNDR is one of the strategic research projects of Saudi Arabia which was approved and funded by King Abdulaziz City for Science and Technology (KACST) and can be accessed at http://www.diabetes.org.sa/diabetes/frames.html by authorized users only. The study was approved by the institutional review board in King Khalid Eye Specialist hospital and the data used in this study was not consented since it does not compromise anonymity or confidentiality or breach of local data protection laws.

A cohort of 67,075 diabetic patients registered between January 2000 and December 2010 was used in this study after completing and validating their data. All patients aged less than 18 years were excluded and a total of 64,351 patients aged ≥ 18 years old were used to assess the prevalence and risk factors associated with III, IV, and VI cranial nerve palsies.

### Study variables

Patients were classified according to their diabetes type into type 1, type 2, impaired glucose tolerance (IGT), and secondary diabetes as per their present status in the registry database and using American Diabetes Association criteria (ADA) [10]. Chronic diabetes complications including: neuropathy in the form of autonomic or polyneuropathy, retinopathy in the form of non-proliferative diabetic retinopathy (NPDR) or proliferative diabetic retinopathy (PDR) with or without macular oedema (ME), nephropathy in the form of microalbuminuria, marcoalbuminurea or end stage renal disease (ESDR) and vasculopathy in the form of cerebrovascular disease (CVD), were recorded if they were documented in the patients’ files.

Patients were classified according to their HbA1c level into well controlled (≤ 7 %) and poorly controlled (> 7 %) using HbA1c value during the last registry visit. Patients were considered hypertensive or hypelipidemic if it was documented in their hospital files or if they were on treatment. All patients using insulin during their registry period were identified as insulin users regardless of their diabetes type.

### Ophthalmoplegia classification

Based on the notes of the ophthalmologist in the patients’ files, ophthalmoplegia was reported when one or more of the following cranial nerves; III, IV, or VI were affected. The oculomotor (III) cranial nerve palsy was considered when it was clearly documented in the patient’s file with one or more of the following signs: droopy lid, restricted upward ocular motility and binocular diplopia [11]. The trochlear (IV) cranial nerve palsy was considered if it was reported that the patient presented with vertical diplopia, which is commonly accompanied by compensatory contra lateral head tilt, while abducens (VI) nerve palsy was considered if the ophthalmologist indicated that the patient had binocular horizontal diplopia and esotropia in primary gaze [12].

All the reported ophthalmoplegic cases were considered to be associated with diabetes if diabetes preceded the onset of ophthalmoplegia and other causes had been excluded by the treating physicians based on the patients’ files review. However, neither the duration nor the recovery status of the reported cases were collected.

### Statistical analysis

The strengthening reporting of observational study in epidemiology (STROBE) guidelines was used to design and report this study. All data were analyzed using Statistical Package for Social Studies (SPSS 22; IBM Corp., New York, NY, USA). Descriptive and frequency analyses were calculated for all variables. The Chi square test (χ2) was used for statistical validation of qualitative variables such as, gender and smoking status. Student’s t-test was used for continuous variables such as age, duration of diabetes BMI and HbA1c. Imputation, based on a regression model, was used to estimate missing data. Risk factors were assessed using univariate, age and gender adjusted, and multivariate logistic regression models. Odds ratio (OR) and its 95 % confidence intervals (CI) were used to express different risks. A p-value of < 0.05 was chosen as the level of significance.

## Results

**Fig. 1:**
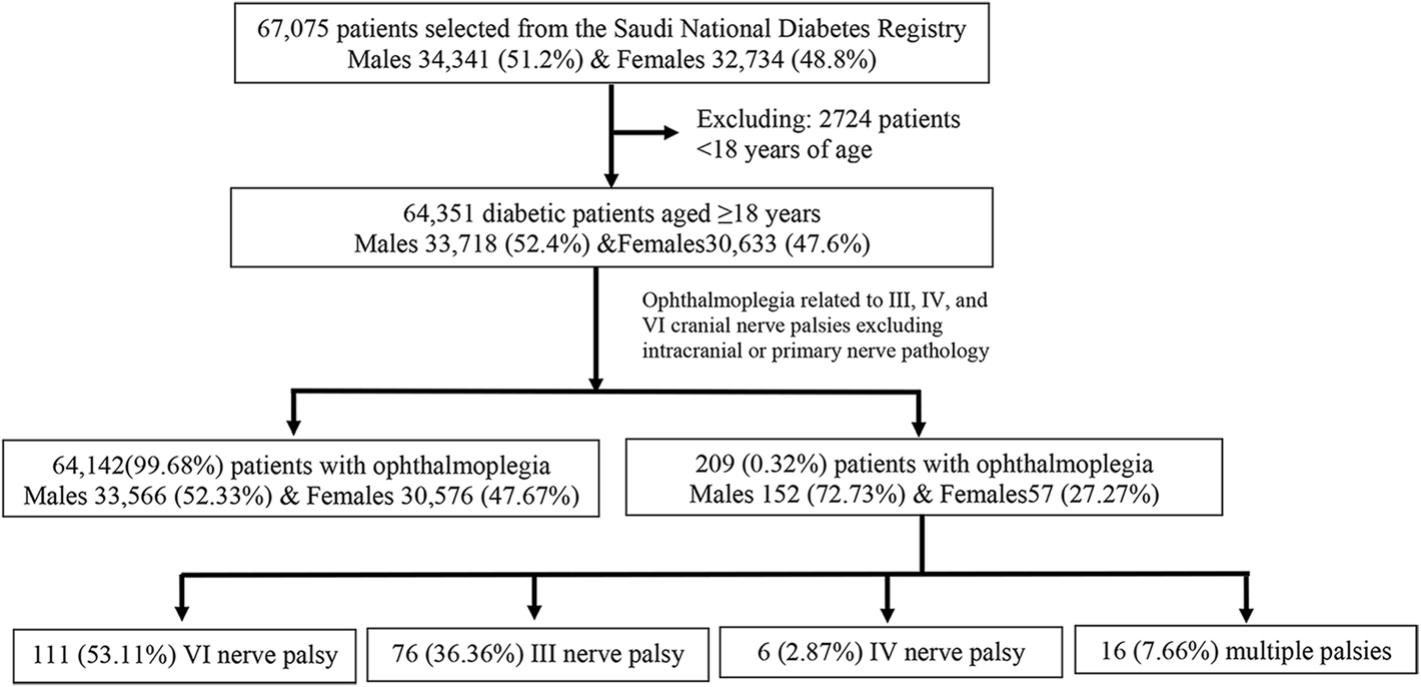
Flow diagram for ophthalmoplegia cases identified among patients ≥ 18 years during the period from 2000 to 2010 using the Saudi National Diabetes registry database

Out of the total studied cohort, 209 (0.32 %) patients were found to be suffering from ophthalmoplegia, where males contributed to 72.73 %, while females contributed to 27.27 % (Fig. 1). A total of 111 patients (53.11 %) had VI cranial never palsy and 76 patients (36.36 %) had III cranial nerve palsy. Patients with IV cranial nerve palsy accounted only for 2.87 % (6 patients), while 16 patients (7.66 %) were suffering from more than one nerve palsy. Ophthalmoplegic patients were significantly older (*p* < 0.0001), having lower body mass index (BMI) (*p* = 0.015) and longer diabetes duration (*p* < 0.0001) when compared with non affected patients. There was no significant difference in the mean HbA1c and fasting blood sugar (FBS) between affected and non-affected patients, while random blood sugar (RBS) was significantly higher among affected cases (Table 1).

**Table 1:** Descriptive analysis of clinical and biochemical characteristics for the patients with or without ophthalmoplegia in the study cohort Sample according to cranial nerve palsy

Variables	Total sample (*n* = 64,351)	Ophthalmoplegia			Cranial nerve palsy			
		Non Affected (*n* = 64,142)	Affected (*n* = 209)	P value	III (*n* = 76)	IV (*n* = 6)	VI (*n* = 111)	Multiple (*n* = 16)
Mean Age	55.92 ± 14.50	55.89 ± 14.51	63.25 ± 9.59	<0.0001	65.17 ± 8.04	64.17 ± 9.11	61.47 ± 9.92	66.13 ± 12.27
Mean BMI	30.47 ± 6.48	30.48 ± 6.48	29.24 ± 5.30	0.015	28.93 ± 5.43	27.20 ± 2.08	29.49 ± 5.28	29.75 ± 6.09
Mean DM duration	13.17 ± 8.05	13.15 ± 8.05	17.90 ± 7.59	<0.0001	17.15 ± 6.86	14.67 ± 4.50	18.20 ± 8.21	20.50 ± 6.93
Mean HbA1c	8.88 ± 2.39	8.88 ± 2.39	8.75 ± 2.19	0.791	9.14 ± 2.32	9.50 ± 0.00	8.45 ± 2.28	8.10 ± 2.12
Mean FBS (mmol/l)	10.02 ± 4.40	10.02 ± 4.40	10.53 ± 4.42	0.402	10.98 ± 4.92	8.60 ± 3.21	10.61 ± 4.35	8.70 ± 0.71
Mean RBS (mmol/l)	12.82 ± 5.63	12.81 ± 5.63	14.87 ± 5.58	0.005	14.52 ± 5.56	15.55 ± 0.21	14.94 ± 5.77	15.59 ± 6.56

Males predominated ophthalmoplegia cases at 72.73 % in both III and VI nerve palsies and all the patients who had IV nerve palsy were males. There were 47.85 % of the ophthalmoplegic patients aged 65 years and older and the rest ( 48.80 %) were between 45 and 64 years of age. We did not report III or IV nerve palsies among those who are younger than 45 years of age. The majority of ophthalmoplegic cases were having BMI more than 25 kg/m^2^.

Type 2 diabetic patients accounted for 99.40 % of all ophthalmoplegic cases found in this study further divided into: 36.71 % with III, 2.90 % with IV, 52.66 % with VI nerves palsies and 7.73 % subjects with multiple cranial nerve involvement. There were only 0.69 % type 1 diabetic patients and all had VI nerve palsy only. There were no cases of ophthalmoplegia found among either IGT or secondary diabetes patients. Over eighty-five percent of the ophthalmoplegic cases had diabetes duration of ≥ 10 years. Smoking was only found in 4.31 % of the affected cases. Out of the total affected cases, 67.94 % had HbA1c > 7 and 36.84 % were insulin users.

Polyneuropathy and autonomic neuropathy affected only 2.87 and 1.91 % respectively of ophthalmoplegic cases. Polyneuropathy was not found to be associated with either IV or III cranial nerves palsies, while autonomic neuropathy was not associated with IV cranial nerve palsy. Ophthalmoplegia was highly associated with NPDR and PDR, with less association with ME at 52.53 %, 47.37 % and 23.92 % respectively. This association was observed for any cranial nerve palsy but not for PDR and ME in IV cranial nerve palsy patients. When compared with other complications, diabetic nephropathy had lower but significant association with ophthalmoplegia, however, no cases with diabetic nephropathy had suffered from IV cranial nerve palsy. Among ophthalmoplegic patients, only 4.31 % reported cerebrovascular disease, which was similar to the non-affected patients. Hypertension was found in 48.33 % of the ophthalmoplegic patients, where approximately 50 % of patients with VI nerve palsy were suffering from hypertension. The presence of hyperlipidemia was significantly lower among ophthalmoplegic patients, when compared with non-affected cases at 24.40 % and 35.42 % respectively (Table 2).

**Table 2:** The number and frequency analysis of the clinical characteristics of patients with and without ophthalmoplegia for the study cohort

Variables		Total sample (*n* = 64,351)	Ophthalmoplegia			Cranial nerve palsy			
			Non Affected (*n* = 64,142)	Affected (*n* = 209)	*P* value	III (*n* = 76)	IV (*n* = 6)	VI (*n* = 111)	Multiple (*n* = 16)
		n (%)	n (%)	n (%)		n (%)	n (%)	n (%)	n (%)
Gender	Male	33,718 (52.40)	33,566 (52.33)	152 (72.73)	< 0.0001	54 (35.53)	6 (3.95)	77 (50.66)	15 (9.87)
	Female	30,633 (47.60)	30,576 (47.67)	57 (27.27)	< 0.0001	22 (38.60)	0 (0.00)	34 (59.65)	1 (1.75)
Age (years)	18-44 years	12,916 (20.07)	12,909 (20.12)	7 (3.35)	< 0.0001	0 (0.00)	0 (0.00)	6 (85.71)	1 (14.29)
	45-64 years	31,914 (49.59)	31,812 (49.60)	102 (48.80)	0.819	34 (33.33)	3 (2.94)	60 (58.82)	5 (4.90)
	≥ 65 years	19,521 (30.34)	19,421 (30.28)	100 (47.85)	< 0.0001	42 (42.00)	3 (3.00)	45 (45.00)	10 (10.00)
Marital status	Single	3,576 (5.55)	3,574 (5.57)	2 (0.96)	0.004	0 (0.00)	0 (0.00)	2 (100.00)	0 (0.00)
	Married	57,567 (89.46)	57,376 (89.45)	191 (91.38)	0.263	71 (37.17)	5 (2.62)	99 (51.83)	16 (8.38)
	Divorced	642 (1.00)	640 (1.00)	2 (0.96)	0.953	0 (0.00)	0 (0.00)	2 (100.00)	0 (0.00)
	Widow	2,566 (3.99)	2,552 (3.98)	14 (6.70)	0.450	5 (35.71)	1 (7.14)	8 (57.14)	0 (0.00)
BMI	18-24.9	11,841 (18.40)	11,797 (18.39)	44 (21.05)	0.322	21 (47.73)	1 (2.27)	19 (43.18)	3 (6.82)
	25-29.9	21,107 (32.80)	21,023 (32.78)	84 (40.19)	0.023	27 (32.14)	5 (5.95)	45 (53.57)	7 (8.33)
	≥ 30	31,403 (48.80)	31,322 (48.83)	81 (38.76)	0.004	28 (34.57)	0 (0.00)	47 (58.02)	6 (7.41)
Type of Diabetes	Type 1	4,368 (6.79)	4,366 (6.81)	2 (0.96)	0.001	0 (0.00)	0 (0.00)	2 (100.00)	0 (0.00)
	Type 2	59,270 (92.11)	59,063 (92.08)	207 (99.04)	< 0.0001	76 (36.71)	6 (2.90)	109 (52.66)	16 (7.73)
	IGT	659 (1.02)	659 (1.03)	0.0 (0.0)	0.284	0	0	0	0
	Secondary DM	54 (0.08)	54 (0.08)	0.0 (0.0)	0.675	0	0	0	0
Diabetes duration	< 10	24,273 (37.72)	24,243 (37.80)	30 (14.35)	< 0.0001	10 (33.33)	1 (3.33)	18 (60.00)	1 (3.33)
	≥ 10	40,078 (62.28)	39,899 (62.20)	179 (85.65)	< 0.0001	66 (36.87)	5 (2.79)	93 (51.96)	15 (8.38)
Smoking		4,183 (6.50)	4,174 (6.51)	9 (4.31)	0.213	3 (33.33)	0 (0.00)	5 (55.56)	1 (11.11)
HbA1c	≤ 7	14,992 (23.30)	14,925 (23.27)	67 (32.06)	0.003	21 (31.34)	2 (2.99)	38 (56.72)	6 (8.95)
	> 7	49,359 (76.70)	49,217 (76.73)	142 (67.94)	0.003	55 (38.73)	4 (2.82)	73 (51.41)	10 (7.04)
Insulin users		19,756 (30.70)	19,679 (30.68)	77 (36.84)	0.053	32 (41.56)	1 (1.30)	37 (48.05)	7 (9.09)
Neuropathy	Poly neuropathy	5,832 (9.06)	5,826 (9.08)	6 (2.87)	0.002	0 (0.00)	0 (0.00)	5 (83.33)	1 (16.67)
	Autonomic neuropathy	1,933 (3.00)	1,929 (3.01)	4 (1.91)	0.538	2 (50.00)	0 (0.00)	2 (50.00)	0 (0.00)
Retinopathy	NPDR	5,123 (7.96)	5,013 (7.82)	110 (52.63)	< 0.0001	36 (32.73)	6 (5.45)	62 (56.36)	6 (5.46)
	PDR	6,146 (9.55)	6,047 (9.43)	99 (47.37)	< 0.0001	40 (40.40)	0 (0.00)	49 (49.50)	10 (10.10)
	ME	2,914 (4.53)	2,864 (4.47)	50 (23.92)	<0.0001	16 (32.00)	0 (0.00)	29 (58.00)	5 (10.00)
Nephropathy		6,280 (9.76)	6,245 (9.74)	35 (16.75)	0.01	11 (31.43)	0 (0.00)	17 (48.57)	7 (20.00)
Cerebrovascular disease		2,817 (4.38)	2,808 (4.38)	9 (4.31)	0.960	3 (33.33)	1 (11.11)	4 (44.45)	1 (11.11)
Hypertension		29,455 (45.77)	29,354 (45.76)	101 (48.33)	0.458	38 (37.62)	1 (0.99)	49 (48.52)	13 (12.87)
Hyperlipidemia		22,767 (35.38)	22,716 (35.42)	51 (24.40)	0.001	22 (43.14)	1 (1.96)	26 (50.98)	2 (3.92)

**Fig. 2:**
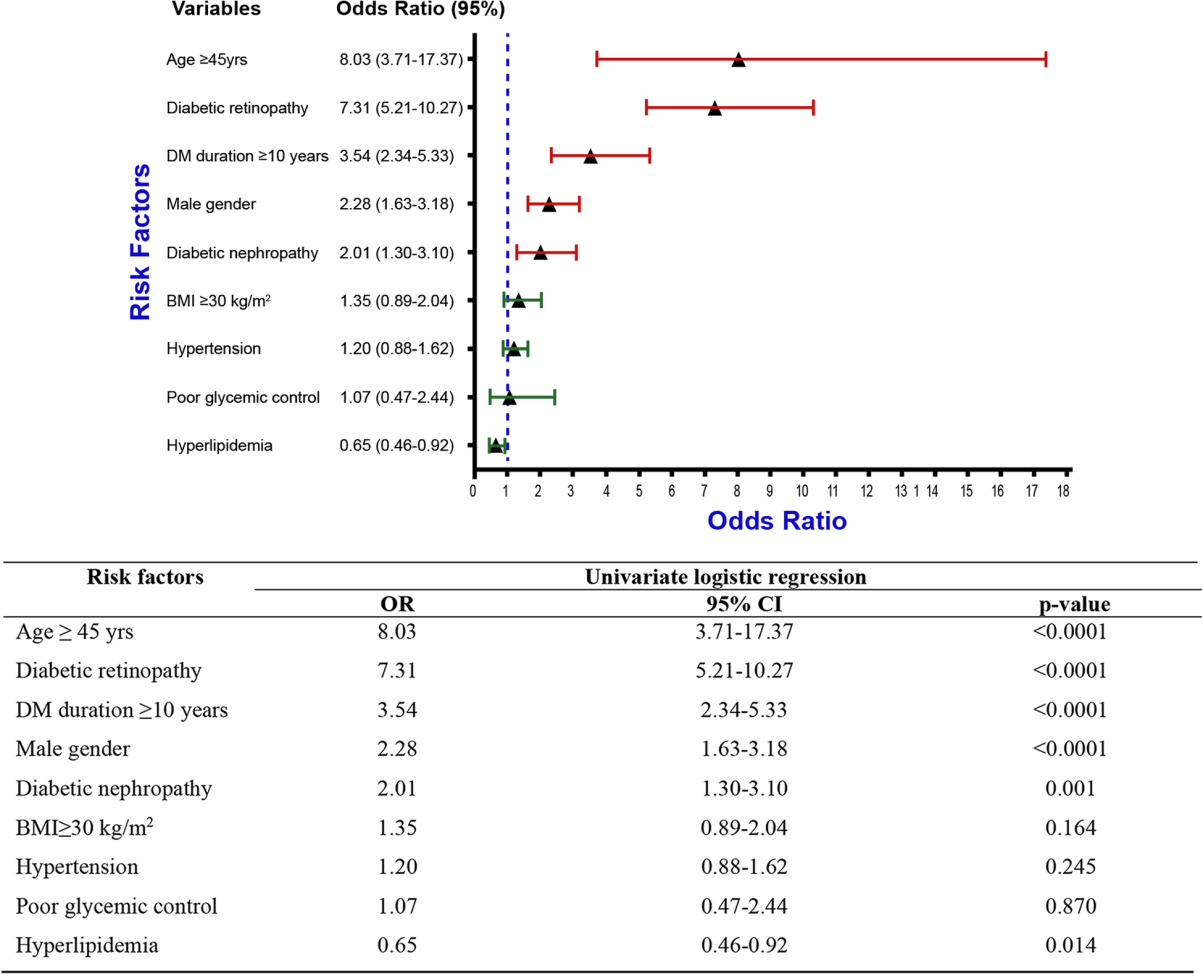
Univariate analysis of the different ophthalmoplegia risk factors

Figure 2 demonstrates the univariate analysis of different risk factors of ophthalmoplegia. Age ≥ 45 years and the presence of diabetic retinopathy were the most important and highly significant risk factors for ophthalmoplegia with OR (95 % CI) of 8.03 (3.71–17.37) and 7.31 (5.21–10.27). Other significant risk factors were diabetes duration ≥ 10 years, male gender, and the presence of diabetic nephropathy with OR (95 % CI) of 3.54 (2.34–5.33), 2.28 (1.63–3.18), and 2.01 (1.30–3.10) respectively. BMI ≥ 30 kg/m^2^, hypertension and poor glycemic control were not found to be significant risk factors, while hyperlipidemia showed a significantly lower risk for ophthalmoplegia.

Table 3 demonstrates the age and gender adjusted in addition to the multivariate regression analysis models for the significant risk factors shown in the univariate analysis. Age and gender adjusted regression analysis demonstrated that, diabetic retinopathy and nephropathy in addition to diabetes duration ≥10 years to be significant and important risk factors. All the five recognized risk factors have proven to be independent when using the multivariate regression analysis, except diabetic nephropathy.

**Table 3:** Age and gender–adjusted, and multivariate–adjusted odds ratio and 95 % confidence intervals of risk factors for the ophthalmoplegia cases in the studied cohort

Risk factors	Age and gender adjusted			Multivariate adjusted		
	OR	95 % CI	*p*-value	OR	95 % CI	*p*-value
Age ≥ 45 years	-	-	-	3.54	1.57–7.97	0.002
Male gender	-	-	-	2.09	1.41–3.08	< 0.0001
Diabetic retinopathy	5.72	4.04–8.11	< 0.0001	4.32	2.94–6.35	< 0.0001
DM duration ≥ 10 years	3.11	2.01–4.80	< 0.0001	1.92	1.19–3.09	0.007
Diabetic nephropathy	1.66	1.06–2.59	0.026	0.86	0.52–1.43	0.570

## Discussion

From our large diabetes registry cohort, this study estimated the prevalence of ophthalmoplegia related to VI, III, and IV cranial nerves palsies to be 0.320 % (0.316–0.324), while it was 0.5 %, 0.4 % and 0.5 % in the Italian studies in 2011 and 2009 and the Japanese study in 1990 respectively [4–6]. Although ophthalmoplegia is a well-known rare entity associated with diabetes, it is reported to be 10 times more frequent than non-diabetic subjects [6]. Cranial nerves involved in ophthalmoplegia had a wide range of frequency as shown in this study, where the most frequent cranial nerve palsy was cranial nerve VI followed by III and IV in the same order. Multiple cranial nerve involvement was found in only 7.6 % of the cases. Although, both the Italian and Japanese studies have shown the third cranial nerve palsy to be the most frequently affected nerve [4, 6], other case report studies from USA, France and UK support our findings that, sixth cranial nerve palsy is the most frequently affected nerve among diabetic patients [13–15]. This was also the same observation reported from a prospective study in a tertiary hospital in Saudi Arabia reported in 1999 [7]. As shown in Table 4, both Italian and Japanese studies did not report any fourth cranial nerve palsy which is known for its very low frequency and diagnosis difficulties. This could be explained by the effect of the small sample which was not the case in our study since we had large cohort and have shown that the fourth cranial nerve palsy frequency to be 2.9 %. This is also supported by findings from France and USA case studies [13, 14], where the large number of ophthalmoplegic cases had shown the frequency of the IV nerve palsy ranging from 6 to 6.7 %. Previous local data have shown fourth nerve palsy frequency to be close to our observation in this study at 2 % [7]. The most likely explanation for the high frequency of VI cranial nerve palsy is related to its significant length [16] which would expose it more to ischemia secondary to angiopathic changes. The frequancy of the involvement of the multiple cranial nerves in our study is similar to what has been reported from other ethnicities except for the Japanese diabetic cohort, where it had reached up to 20 % [6].

**Table 4:** Studies looked at ophthalmoplegic cases in diabetic population

	Study design	Study sample	Number of cases	Prevalence	Cranial nerve palsy			
					VI	III	IV	Multiple
Saudi Arabia (current)	Registry based	64,351	209	0.32 %	53.1	36.4	2.9	7.6
Italy (2011) [1]	Hospital based	8150	61	0.5%^a^	27.3 %	65.9 %	-	6.8 %
Japan (1990) [6]	Hospital based	1961	10	0.5%^a^	20 %	60 %	-	20 %
Italy (2009) [2]	Hospital based	6765	27	0.4 %	29.6	59.3	-	11.1

^a^Prevalence recalculated excluding VII cranial nerve palsy

This study shows that male gender predominated all cases with ophthalmoplegia which was the same observation in Caucasian and Chinese ethnicities [4, 17]. Although ophthalmoplegia is considered to be a condition that affects people after their fifth decade, we are reporting 3.35 % of ophthalmoplegic cases in patients younger than 45 years of age involving mainly cranial nerve VI. The mean BMI for ophthalmoplegic cases in our study demonstrated significantly lower value than non-affected patients although; approximately 80 % of ophthalmologic cases had BMI ≥25 kg/m^2^. We did not report any ophthalmoplegic cases linked to secondary diabetes or IGT patients that could be due to the fact that these two conditions are associated with lower diabetes complications but this observation warrants further studies. Similar to other studies [4, 5], we have also observed that, the vast majority of ophthalmoplegic cases were type 2 diabetic patients, while type 1 was found in less than 1 % of the cases in our study and all were suffering from sixth cranial nerve palsy only. Insulin users were significantly more frequent among ophthalmoplegic patients. This could be explained by the fact that insulin users, especially type 2, had worse diabetes or that insulin has a direct neurotoxic effect [18, 19]. There was no differnce in the frquancy of the cerebrovascular disease between patients with or without ophthalmoplegia.

The most important and highly significant risk factor for ophthalmoplegia in our study was the age ≥ 45 years which was an independent risk factor when multivariate regression analysis was performed which is in consistence with the observation of the vast majority of studies comparing the frequency of this condition in patients before and after 45 years of age [20]. The second important risk factor was the presence of diabetic retinopathy which was also an independent risk factor when adjusting for age and gender or performing multivariate analysis. Diabetic retinopathy either mild (NPDR), or sight threatening (PDR and ME) were significantly more frequent in the ophthalmoplegia cases which is in line with findings of many previous reports [2, 3, 21]. The high frequency of retinopathy among cranial palsy cases could be explained by the fact that both diseases arise from the microvascular abnormalities that are associated with diabetes and its related co-morbid factors [8]. Diabetes duration ≥ 10 years is a significant independent risk factor for ophthalmoplegia among this cohort which was also observed among Caucasian since it was known to be the disease of older age and longer diabetes duration [22]. Similar to other mononeuropathies, male gender in our study was an independent risk factor for ophthalmoplegia wherein males were more frequently affected than females as has been observed by Gaeco D et al. [5] among Caucasian diabetic patients. The current study proves that, diabetic nephropathy is a significant risk factor when univariate and age and gender adjusted regression analysis were conducted, but it was not an independent risk factor for ophthalmoplegia as proven by the multivariate analysis. Although obesity and poor glycemic control were expected to be significant risk factors for ophthalmoplegia, in the current analysis and similar to the findings of Ostricet al., and Kobashi et al. [21, 23], their effect was found to be not significant which could be related to the high frequency of the two factors in both affected and non-affected patients in our cohort. Hypertension did not increase the risk of ophthalmoplegia significantly in the current analysis, which is in line with the findings of Pateland Coll [24] where hypertension was not an independent risk factor. However, it is noteworthy that hypertension may worsen the effect of diabetes in ischemic nerve palsies, especially when the same study reported that the combination of diabetes and hypertension was associated with eightfold increased risk of six nerve palsy [24]. Unexpectedly hyperlipidemia was found to be a significant protective factor with an OR of 0.65 and p value = 0.014. This could be the effect of lipid lowering agents since all hyperlipidemic patients are receiving lipid lowering therapy.

Our study is limited by its hospital-based retrospective nature that lacks certain specific information and with being a cross-sectional study which is not the right set up for determining causality. Another limitation of this study is the lack of temporal relation between HbA1c and the onset of ophthalmoplegia and the possibility of wrong coding or missing any codes.

However, our study draws its strength from being a large diabetes registry cohort that was extensively investigated for diabetes and its co-morbidities.

## Conclusion

Ophthalmoplegia secondary to III, IV and VI cranial nerve palsies among Saudi diabetic patients is a rare entity similar to what has been observed in other ethnicities. This study has shown that age ≥ 45 years, retinopathy, diabetes duration ≥ 10, male gender, and diabetic nephropathy to be the most significant risk factors associated with ophthalmoplegia. We could not find any cases of ophthalmoplegia among patients with secondary diabetes and IGT, and it was even very rare among type 1 diabetic patients. This study provided a better insight on the prevalence of ophthalmoplegia and its different cranial nerves palsies and the important risk factors that clinicians should consider when screening for this rare condition.

## Abbreviations

ADA, American Diabetes Association; CI: confidence intervals; FBS, fasting blood sugar; IGT, impaired glucose tolerance; KACST, King Abdulaziz City for Science and Technology; KFSH, King Faisal Specialist Hospital; ME, macular oedema; NPDR, non-proliferative diabetic retinopathy; OR, odds ratio; PDR, proliferative diabetic retinopathy; RBS, random blood sugar; RC, Research Center; SNDR, Saudi National Diabetes Registry; STROBE, Strengthening Reporting of Observational Study in Epidemiology
